# A new approach to interspecific synchrony in population ecology using tail association

**DOI:** 10.1002/ece3.6732

**Published:** 2020-11-10

**Authors:** Shyamolina Ghosh, Lawrence W. Sheppard, Philip C. Reid, Daniel Reuman

**Affiliations:** ^1^ Department of Ecology and Evolutionary Biology and Kansas Biological Survey University of Kansas Lawrence KS USA; ^2^ Continuous Plankton Recorder Survey The Marine Biological Association, The Laboratory Plymouth UK; ^3^ School of Biological & Marine Sciences University of Plymouth Plymouth UK

**Keywords:** aphids, copula, interspecies synchrony, match–mismatch hypothesis, plankton, tail association

## Abstract

Standard methods for studying the association between two ecologically important variables provide only a small slice of the information content of the association, but statistical approaches are available that provide comprehensive information. In particular, available approaches can reveal *tail associations*, that is, accentuated or reduced associations between the more extreme values of variables. We here study the nature and causes of tail associations between phenological or population‐density variables of co‐located species, and their ecological importance. We employ a simple method of measuring tail associations which we call the *partial Spearman correlation*. Using multidecadal, multi‐species spatiotemporal datasets on aphid first flights and marine phytoplankton population densities, we assess the potential for tail association to illuminate two major topics of study in community ecology: the stability or instability of aggregate community measures such as total community biomass and its relationship with the synchronous or compensatory dynamics of the community's constituent species; and the potential for fluctuations and trends in species phenology to result in trophic mismatches. We find that positively associated fluctuations in the population densities of co‐located species commonly show asymmetric tail associations; that is, it is common for two species’ densities to be more correlated when large than when small, or vice versa. Ordinary measures of association such as correlation do not take this asymmetry into account. Likewise, positively associated fluctuations in the phenology of co‐located species also commonly show asymmetric tail associations. We provide evidence that tail associations between two or more species’ population‐density or phenology time series can be inherited from mutual tail associations of these quantities with an environmental driver. We argue that our understanding of community dynamics and stability, and of phenologies of interacting species, can be meaningfully improved in future work by taking into account tail associations.

## Graphical abstract

We studied the nature and causes of tail associations between phenological or population‐density variables of co‐located species, and their ecological importance to illuminate two major topics of interest in community ecology: the stability or instability of aggregate community measures such as total community biomass and its relationship with the synchronous or compensatory dynamics of the community’s constituent species; and the potential for fluctuations and trends in species phenology to result in trophic mismatches. Standard methods for studying the association between two ecologically important variables provide only a small slice of the information content of the association, whereas we argue that our understanding of community dynamics and stability, and of phenologies of interacting species, can be meaningfully improved in future work by taking into account tail associations.

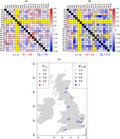

## 1 INTRODUCTION

**Figure 1:**
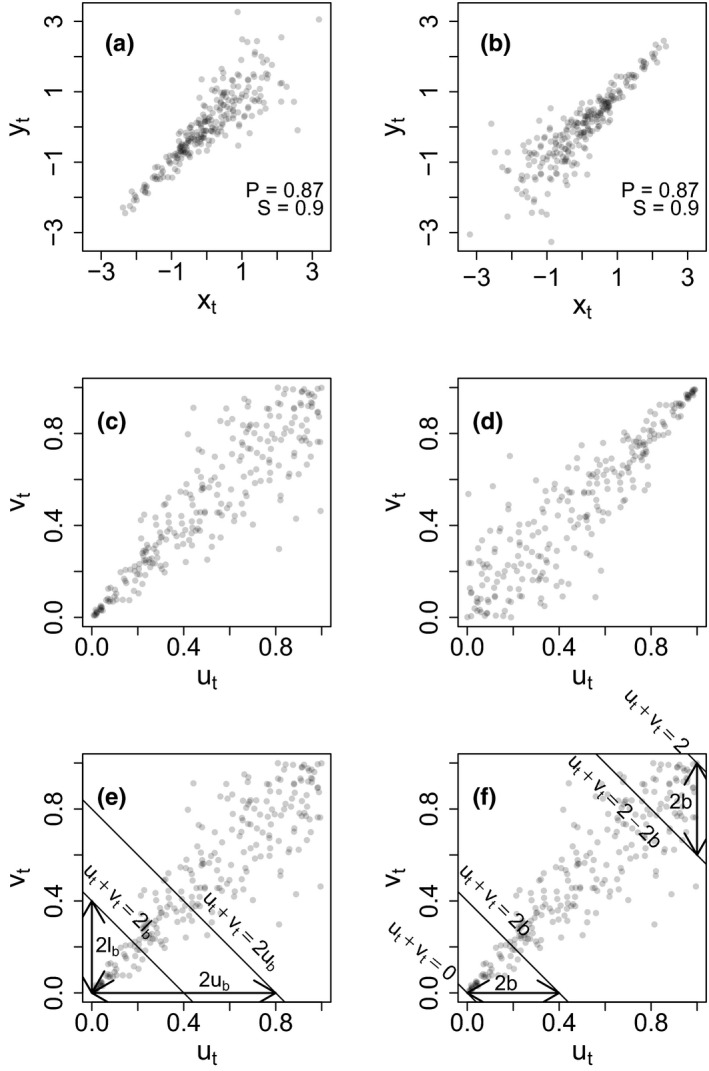
Pedagogical figure for introducing tail association and partial Spearman correlation. (a, b) Two pairs of variables that have identical Pearson (*P*) correlation, and also identical Spearman (S) correlation, but that differ markedly in the nature of the association. Panel a shows stronger left‐ than right‐tail association and panel b shows the reverse. (c, d) Normalized rank plots (see Section 1) for panels a and b, respectively. (e, f) Graphics supporting the definitions of partial Spearman correlation and our statistic measuring asymmetry of tail association (see Section 2). This figure is similar in some respects to figs 1 and 7 of Ghosh, Sheppard, Holder, et al. (2020)

All ecologists study relationships between biological and environmental variables and among biological variables. But standard methods for studying the association between two variables provide only a small slice of the information content of the association. For instance, the two pairs of variables in Figure 1a,b have identical Pearson correlation coefficients, and also have identical Spearman correlation coefficients, but nonetheless display very different patterns of association (Ghosh, Sheppard, Holder, et al., 2020; Ghosh, Sheppard, & Reuman, 2020). Correlations are not the only way to study associations, but they are very commonly used, and other standard methods in ecology provide a similarly limited amount of information that neglects patterns of association (Anderson, de Valpine, Punnett, & Miller, 2018; Genest & Favre, 2007; Joe, 2014; Mai & Scherer, 2017; Nelsen, 2006) that seem likely to be ecologically important (Ghosh, Sheppard, Holder, et al., 2020; Ghosh, Sheppard, & Reuman, 2020).

The variables of Figure 1a (respectively, Figure 1b) are more strongly related in the left (respectively, right) portions of their distributions, thereby displaying asymmetric associations of the distribution tails, henceforth called asymmetric *tail association*. For two positively associated variables, stronger association between values in the left or lower portions of the distributions of the variables is henceforth referred to as *left‐tail association* (Figure 1a), whereas stronger association between values in the right or upper portions of the distributions of the variables is henceforth referred to as *right‐tail association* (Figure 1b). The word “distribution” is sometimes omitted from the terminology, but implied. Tail association is a potentially important pattern of association that is not captured by standard correlation coefficients.

Statistical approaches exist, however, that provides a complete description of the relationship between variables; these approaches are based on the idea of the *copula*. Tail associations are an important aspect of a copula approach to dependence, and tail association will be a focus of this paper. We here give a conceptual flavor of copulas before subsequently focusing on tail association. We introduce copulas instead of proceeding directly to tail associations, for three reasons: to properly credit the copula ideas at the root of our tail association tools, and the researchers who developed them; to indicate the origin of our tail association tools, so that future researchers seeking to generalize our approach will have a place to start; and to introduce ideas (normalized rank plots—see below) that are necessary to define our measures of tail association. Copulas can be used to separate the information content of a bivariate dataset, (*x_t_*, *y_t_*) for *t* = 1,…,*T*, into two nonoverlapping parts: the information in the marginal distributions (which is not about the association between the variables) and the rest of the information (which is solely about the association). Following Ghosh, Sheppard, Holder, et al. (2020) and Genest and Favre (2007), the isolated information about the association between *x_t_* and *y_t_* is revealed by the plot of *u_t_* against *v_t_*, where *u_t_* is the rank of *x_t_* in the set $\{x_{1},x_{2},...,x_{T}\},$ divided by *T* + 1; and *v_t_* is the rank of *y_t_* in the set $\{y_{1},y_{2},...,y_{T}\},$ also divided by *T* + 1. Here the rank of the smallest element of a set is understood to be 1. We refer to the *u_t_* and *v_t_* as *normalized ranks* of the *x_t_* and *y_t_*. We refer to the plot of *v_t_* against *u_t_* as the *normalized rank plot* for *y_t_* and *x_t_*. For instance, the normalized rank plots for Figure 1a,b are in Figure 1c,d and show the asymmetric associations in the tails. The normalized rank plot reflects the copula structure of (*x_t_*, *y_t_*) (Genest & Favre, 2007; Ghosh, Sheppard, Holder, et al., 2020). Ranking makes the marginal distributions uniform, isolating only the information on association between the variables. Genest and Favre (2007) states that inferences about dependence structures should always be based on ranks. It is likewise the purpose of copula approaches to separate association information from information on marginals.

We emphasize that we have not here provided a formal definition of copulas, instead only introducing the fundamental copula idea of separating dependence information from information on marginals. Brief (Anderson et al., 2018; Genest & Favre, 2007; Ghosh, Sheppard, Holder, et al., 2020) and comprehensive (Joe, 2014; Mai & Scherer, 2017; Nelsen, 2006) introductions to copulas are available elsewhere. Copulas can also be used to study multivariate data. Copula approaches are applied widely and to great effect in fields such as finance and neuroscience (Emura & Chen, 2016; Goswami, Hazra, & Goyal, 2018; Kim et al., 2008; Li, 2000; Li, Xie, & Hu, 2013; Onken, Grünwälder, Munk, & Obermayer, 2009; Serinaldi, 2008; She & Xia, 2018), but only rarely, so far, in ecology (Anderson et al., 2018; Ghosh, Sheppard, Holder, et al., 2020; Ghosh, Sheppard, & Reuman, 2020; Popovic, Warton, Thomson, Hui, & Moles, 2019; Valpine, Scranton, Knape, Ram, & Mills, 2014). The potential of copulas for improving ecological understanding was argued by Ghosh, Sheppard, Holder, et al. (2020), and those authors also introduced tail association as an important aspect of copula structure and elaborated the relationship between tail association and copulas.

The study of Ghosh, Sheppard, Holder, et al. (2020) was a wide‐ranging study of the importance, causes, and consequences of copula structures in associations between ecological variables. One of the main foci of that paper was associations between fluctuations through time of population‐density or phenological measurements of the same species in different locations. This study instead focuses on population‐density and phenological measurements of different species in the same location. Ghosh, Sheppard, Holder, et al. (2020) studied, for instance, associations between first flight time series, for a given species of aphid, measured at different locations in the United Kingdom (UK); and associations between plankton density time series, for a given plankton taxon, measured at different locations in seas around the UK. We instead study associations between first flight or population‐density time series measured in the same location for different (sympatric) species. Thus, in contrast with the study of Ghosh, Sheppard, Holder, et al. (2020), this study is more part of community ecology than of spatial ecology. Our reasons for this shift are as follows.

First, *synchronous* (positively correlated) and *compensatory* (negatively correlated) population‐density dynamics of different species occupying the same area are longstanding topics of concern in community ecology, with important ramifications for the stability or instability of aggregate community or ecosystem properties (Gonzalez & Loreau, 2009; Jochimsen, Kümmerlin, & Straile, 2013; Kent, Yannarell, Rusak, Triplett, & McMahon, 2007; Loreau & Mazancourt, 2008; Raimondo, Turcáni, Patoèka, & Liebhold, 2004); there are reasons to believe tail associations in this context will play an important but unstudied role in understanding these topics. A major past insight into community dynamics (Gonzalez & Loreau, 2009) was that an aggregate property of a community, such as its total biomass, can be relatively stable through time although its constituent parts (population biomasses of individual species) are highly variable, if the parts show compensatory dynamics (Hallett et al., 2014). Likewise, synchrony amplifies community biomass variability because the concordant variations of species biomass time series reinforce each other in the total (Ma et al., 2017). If synchronous fluctuations show right‐tail association, then species are highly abundant simultaneously, which may produce years of extremely high community biomass. Alternatively, if synchronous fluctuations show left‐tail association, species are very scarce simultaneously, potentially producing years of extremely low community biomass. Thus the tail association of synchrony, not just the presence and strength of synchrony, may independently influence temporal variability of aggregate community properties. This is revisited in the Discussion.

Second, studies of the phenology of species interacting in one area have also played a central role in community ecology, with important ramifications for whether and to what extent interactions will be modified by climate change (Durant, Hjermann, Ottersen, & Stenseth, 2007; Yang & Rudolf, 2010); there are reasons to believe tail associations between variables in this context may play an important role, as well. As climate changes and phenologies shift, there is the potential for phenologies of interacting species to shift differently, disrupting the interaction (Thackery et al., 2010). This idea is referred to as the match–mismatch hypothesis. Even if, for instance, year‐to‐year fluctuations in the emergence times of two interacting species are highly correlated, if this correlation is principally in the right (respectively, left) tails of the distributions of possible emergence times, so that early (respectively, late) emergences of the species are actually uncorrelated, then mismatched years are likely to occur, impacting the species. Such mismatches will occur, in this conceptual example, when emergence is early (respectively, late). Essentially, even with substantial correlation between emergence dates of species, if this correlation is principally in one of the tails, then uncorrelated emergences, and therefore mismatches, can occur under some conditions. One potential mechanism by which early emergences, for example, may be uncorrelated between species while later emergences remain correlated is if both species follow the same environmental cue for their emergence, but physiological limitations of only one of the species prevent emergence before a certain date. Advancing emergence dates of myriad species make this scenario more plausible.

We here begin exploring whether tail associations may be important for studies of synchrony and compensatory dynamics, and for studies of phenology and the match–mismatch hypothesis. We use a 56‐year dataset of population densities of 4 species of dinoflagellates from the *Certaium* genus, from 15 locations in the seas around the UK; and a 35‐year dataset of annual first flight dates for 20 species of aphid from 10 locations within the UK. The terms left‐ and right‐tail association, defined above, do not apply to negatively associated variables, because the negative association means values in the left tail of one variable are associated with those in the right tail of the other; slightly modified methods are required to study tail association and its asymmetry in negatively associated variables. But our aphid and plankton population and phenology variables were almost exclusively positively associated with each other (see Section 3). Therefore, we introduce methods and present results in this study chiefly for the case of positively associated variables, returning to the topics of negatively associated variables and compensatory population dynamics in the Discussion.

In addition to examining whether tail association in our data is asymmetric, we also test for possible causes of such patterns. One possible mechanism, similar to some of the mechanisms explored by Ghosh, Sheppard, Holder, et al. (2020), is explained for the *Ceratium* example as follows. Earlier work showed that average sea surface temperature is an important correlate of phytoplankton abundance in our data (e.g., Defriez, Sheppard, Reid, & Reuman, 2016; Sheppard, Defriez, Reid, & Reuman, 2019a; Sheppard, Reid, & Reuman, 2017): cold water is associated with more phytoplankton, likely because upwelling and mixing of the surface and deeper ocean layers bring both nutrients and cold water to the photic zone. However, if it is the case for a given location that very cold water is associated with no more *Ceratium*, on average, than is moderately cold water, then that corresponds to a positive relationship and a left‐tail association between the “coldness” of the surface water (measured, for instance, by how many degrees colder the water is than average) and *Ceratium* abundance. If such tail association is strong and consistent across *Ceratium* species, it should produce positive relationships with left‐tail association between the abundance time series of the species. Likewise, in locations for which the winter coldness‐*Ceratium* abundance association shows less left‐tail association, one should see less left‐tail association between different *Ceratium* species. So tail association between two species may be inherited from joint tail association of both species on a common environmental driver. Phytoplankton are also strongly influenced by the abundant generalist copepod consumer *Calanus finmarchicus*, so our actual investigation of the mechanism proposed here will take into account this influence as well as the association with sea surface temperature. For aphid first flight, we examine the same potential mechanism, but the relevant driver in that case is winter temperature.

Thus this paper focuses on whether and why population‐density or phenological time series of co‐located species may show asymmetric patterns in their tail associations, with a focus on positively associated variables because positive associations are what occurred in the available data. We ask the following specific questions. (Q1) Do synchronous/positively correlated population‐density or phenological time series of co‐located species commonly show asymmetric tail associations? (Q2) If so, what are the causes of these patterns? We examine potential ecological consequences of asymmetric tail associations in the Discussion. We regard our investigation as a first step toward a better understanding of the potential importance of asymmetric tail associations for such central ecological topics as synchrony and compensatory dynamics in communities and their influence on community stability; and the match–mismatch hypothesis in phenology. The Discussion also has additional thoughts on next steps toward this goal. Our results and the conceptual considerations introduced above are good evidence, in our view, of the potential for tail association to make a crucial difference in how ecologists understand these important topics.

## 2 METHODS

### 2.1 Data

Our population dataset comprised average annual abundance estimates for 15 locations (Figure S1) in the North Sea and British seas for 4 species from the *Ceratium* genus of dinoflagellates, and for the generalist consumer copepod species *C. finmarchicus*, for the 56 years 1958–2013. These data were a subset of a larger dataset covering 22 taxa and 26 locations, analyzed by Sheppard et al. (2017), Sheppard et al. (2019a), and Ghosh, Sheppard, Holder, et al. (2020). The locations are 2° by 2° grid cells. The data were originally obtained from the Continuous Plankton Recorder (CPR) dataset, now operated and maintained by the Marine Biological Association of the United Kingdom. Data preprocessing steps were the same as used by Ghosh, Sheppard, Holder, et al. (2020). *Ceratium* species were extracted in part because they have a role in harmful algal blooms (red tides) (Baek, Shimode, Shin, Han, & Kikuchi, 2009); and also because four species were available from the genus (Table 1), and we chose closely related species because they may be influenced in similar ways by environmental variables. The 15 locations we used were selected from the 26 locations of the larger dataset (Figure S1) as follows. First, to reduce the effects of sampling variation on statistical results, we chose the subset of locations for which more than 35 years of data were available for all species. Second, for a given location, we excluded *Ceratium* species that were undetected for more than 10% of sampled years at that location. Finally, we considered only those locations for which at least two *Ceratium* species remained. We also had data on average growing season sea surface temperature for each grid cell and year (Sheppard et al., 2017, 2019a). Earlier analyses (e.g., Sheppard et al., 2019a) demonstrated that sea surface temperature and *C. finmarchicus* abundance are important covariates of phytoplankton dynamics in UK seas, though associations between temperature and phytoplankton are probably due to relationships both these variables have with nutrient abundance in surface ocean layers. Sea surface temperature data preprocessing was the same as used by Sheppard et al. (2017).

**Table 1:** Names of 4 plankton and 20 aphid species for which data were used

Species ID	Latin binomial
Plankton	
1	*Ceratium fusus*
2	*Ceratium furca*
3	*Ceratium tripos*
4	*Ceratium macroceros*
Aphids	
1	*Rhopalosiphum insertum*
2	*Rhopalosiphum padi*
3	*Aphis fabae*
4	*Sitobion fragariae*
5	*Hyperomyzus lactucae*
6	*Rhopalosiphum maidis*
7	*Nasonovia ribisnigri*
8	*Phorodon humuli*
9	*Sitobion avenae*
10	*Elatobium abietinum*
11	*Brachycaudus helichrysi*
12	*Brevicoryne brassicae*
13	*Hyalopterus pruni*
14	*Acyrthosiphon pisum*
15	*Myzus persicae*
16	*Macrosiphum euphorbiae*
17	*Metopolophium dirhodum*
18	*Myzus ascalonicus*
19	*Drepanosiphum platanoidis*
20	*Cavariella aegopodii*

Our phenology dataset comprised annual first flight dates for 20 aphid species (Table 1) from 10 locations across the UK (Figure S2), spanning the 35 years 1976–2010. These data were a subset of a larger dataset covering 11 locations, analyzed previously by Sheppard, Bell, Harrington, and Reuman (2016) and Ghosh, Sheppard, Holder, et al. (2020). The data were originally obtained from the Rothamsted Insect Survey suction‐trap dataset (Bell et al., 2015; Harrington, 2014). Data preprocessing was the same as that of Sheppard et al. (2016). Locations were screened, leading to the removal of one of the original 11 sampling locations, by requiring at least 30 years of data be available for all species, again to reduce sampling variation of statistics. We also had time series of winter average temperature for each location and year. The winter temperature for year *t* was the average of December of year *t* − 1 to March of year *t*. Earlier analyses have demonstrated the importance of winter temperature for aphid first flight date (e.g., Sheppard et al., 2016).

### 2.2 Statistical methods

Given bivariate data (*x_t_*, *y_t_*) for a set of years, *t*, of size *T*, and after computing normalized ranks (*u_t_*, *v_t_*) as described in the Introduction, tail association and asymmetry of tail association were measured using the *partial Spearman correlation* of Ghosh, Sheppard, Holder, et al. (2020), which we here reintroduce. The standard Spearman correlation itself measures association between the variables *x_t_* and *y_t_* (or between *u_t_* and *v_t_* – recall the Spearman correlation is based on ranks, so is the same for both sets of variables); but Spearman correlation measures only the overall association of the samples and cannot tell us how association varies across the distributions of the variables. Given two bounds $1\leq l_{b}<u_{b}\leq 1$, we define the boundary lines $u+v=2l_{b}$ and $u+v=2u_{b}$ (Figure 1e), which intersect the unit square on which normalized ranks are plotted. The partial Spearman correlation associated with the bounds *l_b_* and *u_b_* will be the portion of the Spearman correlation attributable to the points that fall between these boundary lines. The partial Spearman correlation for the band between these boundaries and within the unit square is,

(1) $${\text{cor}}_{l_{b},u_{b}}\left(u,v\right)=\frac{\sum \left(u_{t}-\text{mean}\left(u\right)\right)\left(v_{t}-\text{mean}\left(v\right)\right)}{\left(T-1\right)\sqrt{\text{var}(u)\text{var}(v)}}$$

Here, sample means and sample variances are computed using all *T* data points, but the sum, ∑, is over only the indices *t* for which $u_{t}+v_{t}>2l_{b}$ and $u_{t}+v_{t}<2u_{b}.$ The partial Spearman correlation is not defined if there are no points in the band. For positively associated (*u_t_*, *v_t_*), the partial Spearman correlations ${\text{cor}}_{0,b}$ and ${\text{cor}}_{1-b,1}$ for b≤0.5 (Figure 1f) measure association in the left and right tails, respectively, and can be compared via a difference, ${\text{cor}}_{0,b}-{\text{cor}}_{1-b,1}$, to measure asymmetry of tail association. Positive values (respectively, negative) of this difference mean stronger left‐tail (respectively, right‐tail) association. The sum of ${\text{cor}}_{0,0.5}$ and ${\text{cor}}_{0.5,1}$ (or the sum of ${\text{cor}}_{l_{b_{k}},u_{b_{k}}}$ for any other choice of bands $\left(l_{b_{k}},u_{b_{k}}\right)$ that partition (0, 1)) equals the standard Spearman correlation, as long as no points happen to lie exactly on the bounds. Notation is summarized in Table S1.

For each sampling location, *n*, we computed a matrix, $C^{n}$, which we call the *community tail association matrix*, which quantifies asymmetry of tail association between pairs of aphid species or pairs of *Ceratium* species at *n*. Denote by $s_{i}^{n}\left(t\right)$ the aphid first flight date or the *Ceratium* population‐density for sampling location *n*, for the *i*th species that was present in the cleaned data for location *n*, and for year *t*. We then defined the matrix $C^{n}$ by defining $C^{n}\left(i,j\right)$ for two aphid or *Ceratium* species *i*,*j*, as follows. First, $C^{n}\left(i,j\right)$ was not defined, or was defined to equal the missing‐data space holder “NA”, if one of three conditions held true: (a) *i* = *j*; or if (b) the hypothesis that $s_{i}^{n}\left(t\right)$ and $s_{j}^{n}\left(t\right)$ were independent could not be rejected (5% level, using a test described by Genest and Favre (2007), implemented in the function BiCopIndTest in the VineCopula package in R); or if (c) independence was rejected but the Spearman correlation of $s_{i}^{n}\left(t\right)$ and $s_{j}^{n}\left(t\right)$ was negative. Otherwise we defined $C^{n}\left(i,j\right)={\text{cor}}_{0,b}\left(s_{i}^{n}\left(t\right),s_{j}^{n}\left(t\right)\right)-{\text{cor}}_{1-b,1}\left(s_{i}^{n}\left(t\right),s_{j}^{n}\left(t\right)\right)$, where the partial Spearman correlations in this expression were computed over the times, *t*, for which data were available for location *n*. The entry $C^{n}\left(i,j\right)$ was set to NA if independence of $s_{i}^{n}\left(t\right)$ and $s_{j}^{n}\left(t\right)$ could not be rejected because attempting to quantify tail association (or anything else about association) for independent variables is pointless. $C^{n}\left(i,j\right)$ was set to NA for negatively associated $s_{i}^{n}\left(t\right)$ and $s_{j}^{n}\left(t\right)$ because negative association occurred for only one pair of species in one location in our data (plankton sampling location 18, species *C. furca* and *C. macroceros*, see Section 3). Tail association for negatively associated variables should be studied, and this topic is revisited in the Discussion, but negative associations were too rare in our data to study them. The community tail association matrix $C^{n}$ is symmetric. The value b=1/3 was used for plankton locations, whereas b=1/2 was used for aphid locations because aphid time series were shorter, and larger *b* reduces sampling variation for our statistics (Ghosh, Sheppard, Holder, et al., 2020). See Appendix S1 for more information on the choice of *b*.

We also computed a matrix $D^{n}$, which we call the *community‐driver tail association matrix*, which quantifies tail association between aphid or plankton time series and their covariates. Denote by $d_{k}^{n}\left(t\right)$ the value of the *k*th covariate that operated at sampling location *n* in year *t* (winter temperature for an aphid sampling location, sea surface temperature or *C. finmarchicus* density for a *Ceratium* location). We then defined $D^{n}$ by defining $D^{n}\left(i,k\right)$ for an aphid or *Ceratium* species *i* and a covariate *k*, as follows. First, $D^{n}\left(i,k\right)$ was not defined, or was set to NA, if the hypothesis that $s_{i}^{n}\left(t\right)$ and $d_{k}^{n}\left(t\right)$ were independent could not be rejected (5% level, BiCopIndTest). Otherwise, we either: (a) set $D^{n}\left(i,k\right)={\text{cor}}_{0,b}\left(s_{i}^{n}\left(t\right),d_{k}^{n}\left(t\right)\right)-{\text{cor}}_{1-b,1}\left(s_{i}^{n}\left(t\right),d_{k}^{n}\left(t\right)\right)$ if $s_{i}^{n}\left(t\right)$ and $d_{k}^{n}\left(t\right)$ were positively associated (positive Spearman correlation); or (b) set $D^{n}\left(i,k\right)={\text{cor}}_{0,b}\left(s_{i}^{n}\left(t\right),-d_{k}^{n}\left(t\right)\right)-{\text{cor}}_{1-b,1}\left(s_{i}^{n}\left(t\right),-d_{k}^{n}\left(t\right)\right)$ if $s_{i}^{n}\left(t\right)$ and $d_{k}^{n}\left(t\right)$ were negatively associated (negative Spearman correlation). For aphid first flight time series, for which *k* was always 1 and $d_{k}^{n}\left(t\right)$ was winter temperature in location *n*, associations between $s_{i}^{n}\left(t\right)$ and $d_{k}^{n}\left(t\right)$ were always negative when they were significant (see Section 3). The same was true for *Ceratium* density time series and sea surface temperature. Thus our practice of using $-d_{k}^{n}\left(t\right)$ was equivalent, in the case of temperature variables, to using a “coldness” index such as the number of degrees colder than an average or typical reference temperature, in place of temperature. Aphid and *Ceratium* data were always positively associated with the coldness index when they were significantly associated with it. Although *C. finmarchicus* abundance was positively associated with *Ceratium* time series in some sampling locations and negatively associated in others, it always showed the same sign of association with all *Ceratium* species within a location. Using $-d_{k}^{n}\left(t\right)$ in place of $d_{k}^{n}\left(t\right)$ when negative associations with aphid or *Ceratium* data occurred allowed us to study asymmetry of tail association using methods developed with positively associated variables in mind. We again used b=1/3 for plankton data and covariates, and b=1/2 for aphid data and winter temperature. For display, we horizontally concatenated the matrices $C^{n}$ and $D^{n}$ and displayed matrix values using color.

We used the community tail association matrix $C^{n}$ for each sampling location *n* to answer Q1 from the Introduction, as follows. First, we counted the number, $N_{L}^{n}$, of entries of $C^{n}$ which were not NA and which were greater than 0. These were the “left‐tail dominant” species pairs, that is, pairs of species for which association was stronger in the left rather than in the right tails of the species distributions. We also counted the number, $N_{R}^{n}$, of right‐tail dominant pairs, for which the corresponding entries of $C^{n}$ were negative. If $N_{L}^{n}$ was substantially greater than (respectively, substantially less than) $N_{R}^{n}$ for a location *n*, it suggested that left‐tail association (respectively, right‐tail association) between species in that location was dominant, answering Q1 in the affirmative. We also calculated $A_{C,L}^{n}$, the sum of all positive, non‐NA entries of $C^{n}$; $A_{C,R}^{n}$, the sum of all negative, non‐NA entries of $C^{n}$; and $A_{C}^{n}=A_{C,L}^{n}+A_{C,R}^{n}$, a general measure of asymmetry of tail association in location *n*. We refer to $A_{C}^{n}$ as the *total community tail association*. We additionally calculated the normalized quantities $F_{C,L}^{n}=A_{C,L}^{n}/\left(A_{C,L}^{n}+|A_{C,R}^{n}|\right)$ and $F_{C,R}^{n}=A_{C,R}^{n}/\left(A_{C,L}^{n}+|A_{C,R}^{n}|\right)$. Because $0\leq F_{C,L}^{n}\leq 1$, $0\leq |F_{C,R}^{n}|\leq 1$, and $F_{C,L}^{n}+\left|F_{C,R}^{n}\right|=1$, the relative sizes of $F_{C,L}^{n}$ and $\left|F_{C,R}^{n}\right|$ indicate the relative dominance of left‐ and right‐tail association between species at location *n*. Together, all these statistics provide an answer to Q1.

We used the community tail association matrix, $C^{n}$, and the community‐driver tail association matrix, $D^{n}$, to answer Q2 from the Introduction for the *Ceratium* and aphid data, as follows. First, we calculated $A_{D}^{n}$, the sum of all non‐NA entries of $D^{n}$. This was analogous to $A_{C}^{n}$, but calculated using the matrix $D^{n}$ instead of the matrix $C^{n}$. We refer to $A_{D}^{n}$ as the *total community‐driver tail association*. We then examined whether the values $A_{C}^{n}$ and $A_{D}^{n}$ were correlated across locations, *n*. This tests the causal hypothesis in the Introduction because it tests whether *Ceratium* or aphid time series having stronger right‐tail (respectively, left‐tail) association with environmental covariates in a given location also had stronger right‐tail (respectively, left‐tail) association with each other at that location. Recall that an environmental covariate was reversed (its negative was used) when it was negatively associated with a *Ceratium* or aphid species, and that no covariate was ever significantly positively associated with some *Ceratium* or aphid species and significantly negatively associated with another such species in the same location (see Section 3).

We also answered Q2 for the aphid data as follows. Within a location, *n*, for each species, *i*, we computed the mean $\alpha_{C}^{n}\left(i\right)$ of all non‐NA entries $C^{n}\left(i,j\right)$, for *j* ranging across all species for which we had data. This quantity measures an average tail association of species *i* with other species in the same location, with positive values for greater left‐tail association and negative ones for greater right‐tail association. We refer to $\alpha_{C}^{n}\left(i\right)$ as the *species‐community tail association* for species i. We then defined $\alpha_{D}^{n}\left(i\right)$ as the sum of all non‐NA entries $D^{n}\left(i,k\right),$ for *k* ranging across all covariates for which we had data. We refer to this as the *species‐driver tail association* for species *i*. For aphids we only had one covariate, winter temperature, so $\alpha_{D}^{n}\left(i\right)=D^{n}\left(i,k\right)$ for *k* = 1 corresponding to winter temperature. We provide the more general definition of $\alpha_{D}^{n}\left(i\right)$ that applies when more covariates were available so the definition can also be considered (briefly, see below) for *Ceratium* data. We then examined, for each location, *n*, whether $\alpha_{C}^{n}\left(i\right)$ and $\alpha_{D}^{n}\left(i\right)$ were correlated across species, *i*. This tests the causal hypothesis in the Introduction because it tests whether aphid species which were more right‐tail (respectively, left‐tail) associated with environmental covariates (winter temperature) also had time series that were more right‐tail (respectively, left‐tail) associated with the time series of other species in the location. Recall that winter temperature was always negatively associated with aphid first flight when it was significantly associated (see Section 3), and negative temperature (a coldness index) was used in computing $D^{n}\left(i,k\right)$. Testing whether $\alpha_{C}^{n}\left(i\right)$ and $\alpha_{D}^{n}\left(i\right)$ were correlated across species, *i*, within a location, *n*, was not practical for *Ceratium*, because we only had data for at most four *Ceratium* species per sampling location, an insufficient number to provide much statistical power in testing for a correlation.

## 3 RESULTS

**Figure 2:**
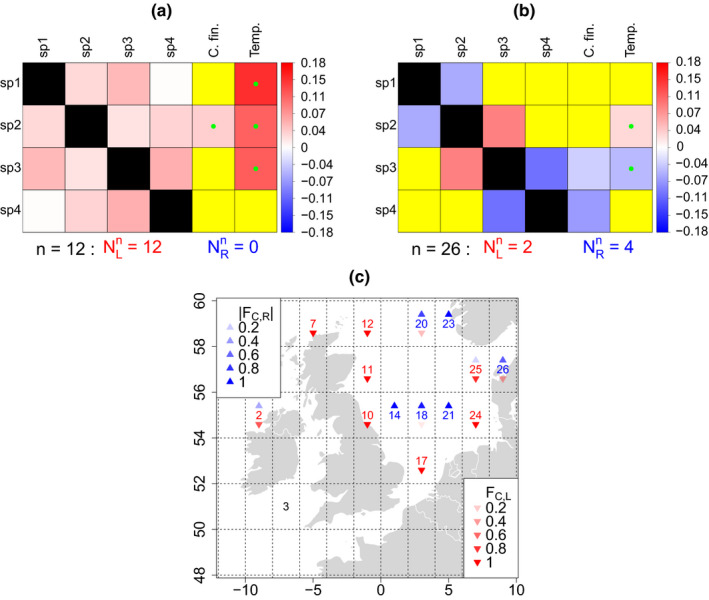
Either right‐ or left‐tail association between population‐density time series of *Ceratium* species could dominate, depending on the sampling location. (a, b) The community tail association matrix, $C^{n}$, and the community‐driver tail association matrix, $D^{n}$ (Statistical methods), horizontally concatenated, for example locations *n* = 12 (a) and *n* = 26 (b). See Table 1 for species names. All the non‐NA values in $C^{n}$ were positive (red) for location 12 (a), indicating left‐tail association dominated in that location; but values were largely negative (blue) for location 26 (b), indicating right‐tail association dominated there. Matrix entries which were NA because time series were independent are displayed in yellow. The counts $N_{L}^{n}$ and $N_{R}^{n}$ (see Section 2.2) also reflect the distinct tail association characteristics of the two locations. *C. fin.* = *C. finmarchicus*; Temp. = temperature. Green dots in $D^{n}$ represent variables which were originally negatively associated, so the negative of the environmental covariate was used for calculating tail association. See Figure S3 for analogous figures for the other sampling locations. (c) The summary statistics $F_{C,L}$ and $F_{C,R}$ (see Section 2.2) for each site show that association between *Ceratium* species was either substantially dominated by the left or right tails of *Ceratium* distributions, with the exceptions of a few locations for which tail association was closer to symmetric. Site codes are colored red or blue depending on which of $F_{C,L}$ or $F_{C,R}$ had higher magnitude. Values are not plotted for site 3 because the hypothesis could not be rejected for that site that dynamics of distinct *Ceratium* species were independent

Associations between *Ceratium* species were always positive when they were significant, except for one pair of species in one location (plankton sampling location 18, species *C. furca* and *C. macroceros*). Asymmetric tail association was very common between *Ceratium* population‐density time series from the same location, answering Q1 in the affirmative for *Ceratium*; for some locations, left‐tail association between *Ceratium* species was dominant, and for other locations right‐tail association was dominant. To show this, we show that for some locations, the community tail association matrix, $C^{n},$ was comprised largely of positive values, indicating a preponderance of left‐tail association between *Ceratium* time series for the location (Figure 2a). For such locations, *Ceratium* population densities are more likely to be correlated across species at low population densities than at high densities. For other locations, $C^{n}$ had mostly negative values, indicating a preponderance of right‐tail association (Figure 2b). For such locations, *Ceratium* population densities are more likely to be correlated across species at high population densities than at low densities. To demonstrate the same result in another way, we show that the statistics $F_{C,L}$ and $F_{C,R}$, plotted across all sampling locations (Figure 2c), indicated that most *Ceratium* sampling locations were dominated by either left‐ or right‐tail association, with approximately equal numbers of each, with only a few locations having more symmetric tail association, on average across pairs of *Ceratium* species.

**Figure 3:**
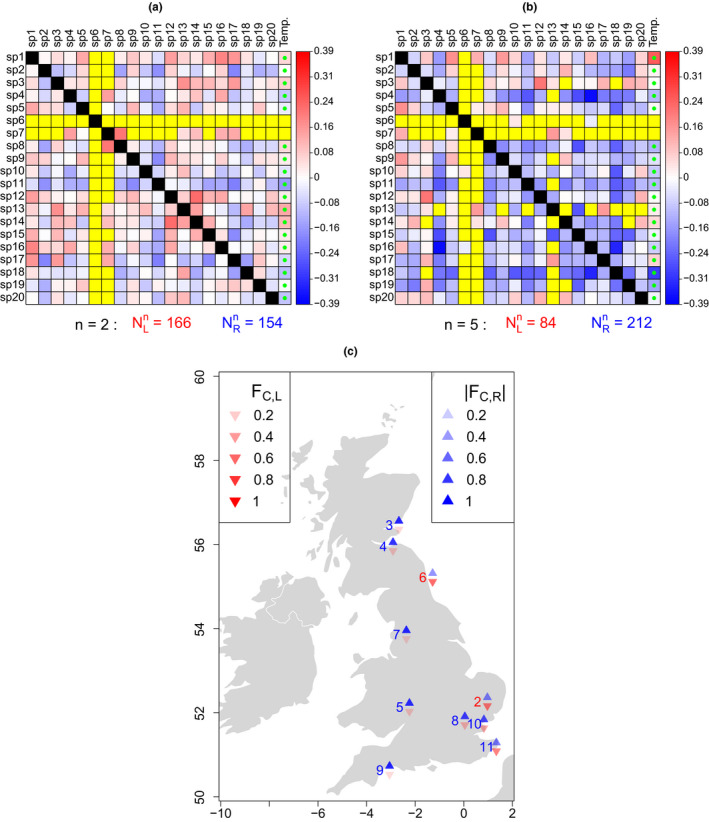
Either right‐tail association between first flight time series of aphid species could dominate, or left‐tail association could be more common, depending on the sampling location. (a, b) The community tail association matrix, $C^{n}$, and the community‐driver tail association matrix, $D^{n}$ (Statistical methods), horizontally concatenated, for example locations *n* = 2 (a) and *n* = 5 (b). See Table 1 for species names. A slight majority of non‐NA values in $C^{n}$ were positive (red) for location 2 (a; see the $N_{L}^{n}$ and $N_{R}^{n}$ counts displayed), indicating left‐tail association was slightly more common than right‐tail association in that location. But values were largely negative (blue) for location 5 (b), indicating right‐tail association dominated there. Matrix entries which were NA because time series were independent are displayed in yellow. Temp. = temperature. Green dots in $D^{n}$ represent variables which were originally negatively associated, so the negative of winter temperature was used for calculating tail association (Statistical methods); this happened in all cases for which temperature and first flight were significantly associated. See Figure S4 for analogous figures for the other sampling locations. (c) The summary statistics $F_{C,L}$ and $F_{C,R}$ (see Section 2.2) for each site show that association was either dominated by the right tails, or, for a few locations, showed slightly more left‐tail association. Site codes are colored red or blue depending on which of $F_{C,L}$ or $F_{C,R}$ had higher magnitude

Associations between aphid time series were always positive when they were significant. Asymmetric tail association was also very common between aphid first flight time series from the same location, answering Q1 in the affirmative for aphids; left‐tail association was more common for some sampling locations and right‐tail association dominated for others, but for most sites right‐tail association dominated. To show this, we show that for some locations, the community tail association matrix, $C^{n}$, was comprised of a slight majority of positive values, indicating more left‐ than right‐tail association between aphid time series for the location (Figure 3a); whereas for other locations, $C^{n}$ had mostly negative values, indicating a preponderance of right‐tail association (Figure 3b). To demonstrate the same result in another way, we show that the statistics $F_{C,L}$ and $F_{C,R}$, plotted across all sampling locations (Figure 3c), indicated that most aphid sampling locations had a preponderance of right‐tail association, with only a few locations having more left‐tail association, and those only slightly more. Thus, for most locations, aphid first flights are more correlated across species when first flights are later than average.

**Figure 4:**
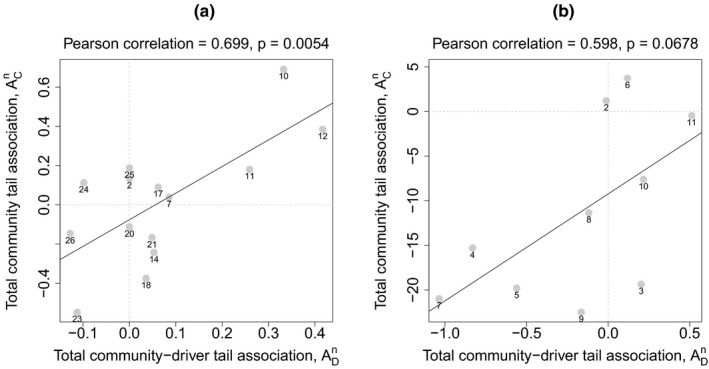
Tail association with environmental covariates was positively related to tail association between species for aphid and plankton time series. Panels show total community tail association, $A_{C}^{n},$ plotted against total community‐driver tail association, $A_{D}^{n}$ (Statistical methods), across locations, *n*, for *Ceratium* density (a) and aphid first flight (b) data. Pearson correlations and associated *p*‐values for each panel are in the headers. Points are labeled with location numbers (see Figures S1 and S2)

For the *Ceratium* data, the total community tail association, $A_{C}^{n}$, and the total community‐driver tail association, $A_{D}^{n}$, were significantly correlated across locations, *n*, validating our hypothesis from the Introduction for a cause of tail association between co‐located species, and helping to answer Q2. In other words, tail association between co‐located species time series was apparently inherited from common tail association of the species on environmental drivers. Across our 15 locations, $A_{C}^{n}$ and $A_{D}^{n}$ were significantly positively correlated (Pearson correlation, two‐tailed test, Figure 4a). Thus locations for which *Ceratium* density time series showed greater left‐tail (respectively, right‐tail) association with environmental covariates (measured with $A_{D}^{n}$) also exhibited greater left‐tail (respectively, right‐tail) association between density time series for distinct species (measured with $A_{C}^{n}$).

For the aphid data, the total community tail association, $A_{C}^{n},$ and the total community‐driver tail association, $A_{D}^{n},$ were positively but nonsignificantly correlated across our 10 sampling locations (Figure 4b). Thus locations for which aphid first flight time series showed greater left‐tail (respectively, right‐tail) association with winter temperature also showed a nonsignificant tendency toward greater left‐tail (respectively, right‐tail) association between the time series of distinct species. The correlation was close to significant for the aphid data, and may have been nonsignificant simply because there were slightly fewer aphid sampling locations than there were plankton locations. See also the subsequent results for aphids, which were significant and which support the same overall conclusions.

**Figure 5:**
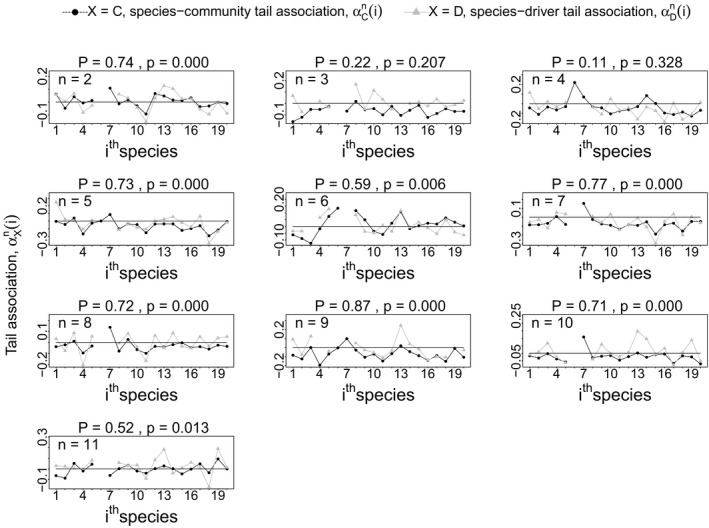
For 8 out of 10 sites, the Pearson correlation (*P*) between the species‐community tail association, $\alpha_{C}^{n}\left(i\right)$, and the species‐driver tail association, $\alpha_{D}^{n}\left(i\right)$, across *i* = 1, 2, …, 20, was significantly positive (*p* < .05, one tailed test). This supports the hypothesis that tail association between species may be inherited from joint tail association of both species on a common environmental driver. See Table 1 for species IDs

Our second analysis using aphids, based on the species‐community tail associations, $\alpha_{C}^{n}\left(i\right),$ and the species‐driver tail associations, $\alpha_{D}^{n}\left(i\right)$ (Statistical methods), provided further evidence supporting our hypothesis for a cause of tail association between co‐located species (Introduction). For 8 of 10 sampling locations, $\alpha_{C}^{n}\left(i\right)$ and $\alpha_{D}^{n}\left(i\right)$ were significantly correlated across species, *i* (Figure 5). In other words, for 8 of 10 locations, aphid species with greater left‐tail (respectively, right‐tail) association with winter temperature also had greater left‐tail (respectively, right‐tail) association with other aphid species.

## 4 DISCUSSION

Our results show that synchronous population‐density or phenological time series of co‐located species can very commonly show asymmetric tail association. For some sampling locations and species, tail association was predominantly in the left tails, and for others it was predominantly in the right tails of time series distributions, showing a new kind of ecologically meaningful variation among ecosystems. The partial Spearman correlation presented by Ghosh, Sheppard, Holder, et al. (2020) is a simple and effective way to measure tail association for ecological applications. Our results also demonstrate a mechanism by which asymmetric tail association between species can arise: It can be inherited by joint tail association of the two species on the same environmental variables. This mechanism seems likely to apply commonly when co‐located species are influenced by the same external factors. Our results convincingly show that standard correlation approaches omit phenomena that seem likely to be important for at least two major topics of interest in ecology: synchronous/compensatory dynamics of species within a community and their influence on community stability; and shifting phenologies and the match–mismatch hypothesis.

The distinct tail association characteristics of *Ceratium* in different sampling areas around the UK may have consequences for the stability through time of total *Ceratium* abundance, which may relate to harmful algal blooms because *Ceratium* species can have a role in such blooms (Baek et al., 2009). For locations in which left‐tail association between *Ceratium* density time series is dominant, *Ceratium* species are scarce simultaneously, potentially producing years of very low total *Ceratium* biomass. In contrast, for locations in which right‐tail association is dominant, *Ceratium* species are highly abundant simultaneously, which may produce years of very high *Ceratium* biomass, which may sometimes correspond to harmful algal blooms. Our results show that the distinction between these two types of location relates to the tail association of *Ceratium* species with their environmental covariates, sea surface temperature and *C. finmarchicus* density. It may be useful to study in future work why some locations principally have left‐tail association with these drivers and some principally have right‐tail association.

First flight time series for populations of co‐located aphid species were principally right‐tail associated; that is, more strongly correlated when first flights were later in the season. Our results show this was probably because: cold winters delay aphid first flights, but warm winters do not lead to first flights that are any earlier, on average, than those following moderate winters, producing right‐tail association between first flights and winter coldness across multiple species; this common association leads to right‐tail association between aphids. Thus winter temperature fluctuations lead to temporally dispersed early but temporally coordinated late arrival times of aphid species on summer hosts (many of which are crops, for the species we studied), a fact that may have pest‐control significance. Winter temperature is known to influence the first flight dates of virtually all the aphid species for which we had data (Sheppard et al., 2016). Overwintering aphids are sensitive to frost conditions, and so winters probably reduce early spring populations on winter hosts plants. This then lengthens the time required for populations to reach sufficient densities to stimulate the production of winged morphs for flight to summer host plants.

If $x_{s,l}\left(t\right)$ denotes the population‐density of species s(s=1,...,S) in location l(l=1,...,L) at time *t*, we have here studied the nature and causes of tail association among the time series $x_{s,l}\left(t\right)$ for a fixed *l* and for s=1,...,S; whereas Ghosh, Sheppard, Holder, et al. (2020) studied the nature, causes and consequences of tail association among the time series $x_{s,l}\left(t\right)$ for fixed *s* and l=1,...,L, a distinct ecological context. One of the consequences studied by Ghosh, Sheppard, Holder, et al. (2020) relates to and illuminates a potential consequence, mentioned above, of tail association for the ecological context of this study. Ghosh, Sheppard, Holder, et al. (2020) showed that the skewness, though time, of the spatial‐total time series $\sum_{l}x_{s,l}\left(t\right)$ is sensitive to the nature of tail association between the $x_{s,l}\left(t\right)\;\left(l=1,...,L\right)$, if these time series are positively associated with each other. Right‐tail (respectively, left‐tail) association tended to produce right (respectively, left) skew in the total. Right skew corresponds to a spatial‐total time series with exceptionally large values, that is, to “spiky”, unstable dynamics of the total population. Left skew corresponds to a spatial‐total time series with low values, that is, to dynamics of the total population with a tendency to “crash”. The total population can be regarded as a landscape‐level measure of the stability or variability of species *s*, and is important, for instance, if species *s* is a pest or an exploited species. For the same reasons, the skewness, through time, of the community‐total time series $\sum_{s}x_{s,l}\left(t\right)$ is sensitive to the tail association between the $x_{s,l}\left(t\right)\;\left(s=1,...,S\right)$, which we have here studied. Right‐tail (respectively, left‐tail) association again tends to produce right (respectively, left) skew in the total time series. In this community context, the total is an aggregate property of the community, and the variability of this total has been used in an extensive literature (e.g., Hallett et al., 2014) to characterize community stability through time. This literature has explored the effects of synchronous versus compensatory dynamics in the $x_{s,l}\left(t\right)\;\left(s=1,...,S\right)$ on the stability of the total community time series, $\sum_{s}x_{s,l}\left(t\right)$. But our results show that, even if all the species time series $x_{s,l}\left(t\right)\;\left(s=1,...,S\right)$ are synchronous with each other, the tail association properties of these time series can influence the stability of the community‐total time series.

Although our results are sufficient to show that tail associations are likely to be important for studies of community dynamics and stability, many communities show not only synchronous dynamics between some species pairs $x_{s_{i},l}\left(t\right)$ and $x_{s_{j},l}\left(t\right)$, but also compensatory dynamics between other pairs. Our *Ceratium* time series were almost entirely synchronous, so we could not study the importance of tail association for compensatory dynamics. Next research steps should include the study of tail association between compensatory species within a local community. Furthermore, *Ceratium is* only part of the phytoplankton community in UK seas. It may be advantageous for future work to use data characterizing an entire competitive community. For instance, the data of Hallett et al. (2014) constitute annual abundances of all species of plant in an area. In that dataset, some species pairs show synchronous and some show compensatory dynamics.

Studying asymmetry of tail association for negatively correlated species density time series will require slightly modified methods. The only negative association between aphid or *Ceratium* time series that occurred in our system was not analyzed. Negative associations between species time series and the environmental covariates we considered were handled statistically by considering the positive association between the species time series and a “reversed” covariate; this corresponds to a positive association with a reconceptualized covariate, for example, a “coldness” index. But that approach would make no sense for negatively associated time series of two aphid or *Ceratium* time series: there is no canonical choice of which variable to reverse. Asymmetry of tail association could still be considered, however, for negatively associated variables, *u*, *v*, in an unsigned approach, via the index $|{\text{cor}}_{0,b}\left(u,1-v\right)-{\text{cor}}_{1-b,1}\left(u,1-v\right)|$. Because $|{\text{cor}}_{0,b}\left(u,1-v\right)-{\text{cor}}_{1-b,1}\left(u,1-v\right)|\;=\;|{\text{cor}}_{0,b}\left(1-u,v\right)-{\text{cor}}_{1-b,1}\left(1-u,v\right)|$ no choice need be made on which variable to “reverse.” A large value of this index indicates that tail association between *u* and *v* is asymmetric, though it does not provide information on whether association is stronger between the left tail of *u* and the right tail of *v* or between the right tail of *u* and the left tail of *v*.

Measures of tail association may also reveal useful information about freshwater plankton ecosystems and harmful algal blooms, in addition to information about marine harmful algal blooms (discussed above). Because blooms are extreme phenomena involving multiple species, monitoring the associations of phytoplankton species with each other and their associations with temperature and nutrient data in the extremes (this is tail association) could help us to better understand harmful blooms. Considering tail association may even produce improvements in statistics that have been developed to serve as early warning signals of impending major changes (so‐called “tipping points”) in plankton communities and the lakes they inhabit (Butitta, Carpenter, Loken, Pace, & Stanley, 2017; Carpenter et al., 2011), since some established early warning statistics make use of skewness of population distributions (Guttal & Jayaprakash, 2008). Tail association between phytoplankton species is related to skewness of the total phytoplankton biomass time series, as described in an earlier Discussion paragraph.

Although our aphid results were sufficient to demonstrate that tail association can be an important factor in the phenology of co‐located species, it will be necessary in future work to apply tail association ideas to different datasets to assess whether these ideas can improve our understanding of the consequences of changing phenology for trophic phenological matching. The aphid species we studied have different host plants, so they do not directly interact. Shifts and fluctuations in the phenology of one species probably do not directly influence other species in our dataset. Future research should apply tail association to time series of phenologies of interacting species, such as the data on tree budburst dates, caterpillar abundance, and breeding phenology of great tits (*Parsus major*) and blue tits (*P. caeruleus*) collected in Wytham Woods, Oxford, and other locations in Europe (e.g., Cole & Sheldon, 2017; Nilsson & Källander, 2006; Savill, Perrins, Kirby, & Fisher, 2011), or the extensive data collection from multiple trophic levels of Thackery et al. (2010).

One final idea for potentially valuable future research has to do with combining our approach, based on tail associations, with other recent approaches which emphasize other statistical aspects of the synchrony. For instance, research has now showed that synchrony and compensatory dynamics in communities have “timescale structure”; that is, the dynamics of two or more species can be synchronous on some timescales of analysis and compensatory on others (Keitt & Fisher, 2006; Vasseur et al., 2015; Zhao et al., 2020). How timescale specificity and tail associations interact is unknown, but potentially interesting. Multivariate copula approaches (Czado, 2019; Joe, 2014) may be useful in this and other future extensions of the work we have begun here.

Our results extend the results of Ghosh, Sheppard, Holder, et al. (2020). Those authors argued that considering copulas and tail associations can provide insights across the field of ecology. But Ghosh, Sheppard, Holder, et al. (2020) did not consider co‐located species, a context important for community ecology which we considered here.

## CONFLICT OF INTEREST

The authors declare no conflict of interest.

## AUTHOR CONTRIBUTIONS

**Shyamolina Ghosh:** Conceptualization (equal); Formal analysis (lead); Methodology (equal); Software (lead); Visualization (lead); Writing‐original draft (lead); Writing‐review & editing (equal). **Lawrence W. Sheppard:** Conceptualization (equal); Formal analysis (supporting); Writing‐review & editing (supporting). **Philip C. Reid:** Data curation (lead); Writing‐review & editing (supporting). **Daniel C Reuman:** Conceptualization (equal); Formal analysis (supporting); Funding acquisition (lead); Methodology (equal); Supervision (lead); Writing‐original draft (equal); Writing‐review & editing (equal).

## Supporting information

Supplementary Material

## Data Availability

Plankton data are available from the Dryad Digital Repository https://doi.org/10.5061/dryad.rq3jc84 (Sheppard, Defriez, Reid, & Reuman, 2019b). Full code for the analyses can be downloaded from Dryad Digital Repository https://datadryad.org/stash/share/eC20ojo_e9UTmXAoX1oq3kIfT3aY0iptcUqAk8MHrAA.
