# Supplementary material for: A new approach to interspecific synchrony in population ecology using tail association
Source: Ecol Evol. 2020 Nov 10;10(23):12764–76. doi: 10.1002/ece3.6732 (PMC7713959; doi:10.1002/ece3.6732)
Supplement: Supplementary file 1 — Supplementary Material [file ECE3-10-12764-s001.pdf]

Supporting material: A new approach to interspecific synchrony in population ecology using tail association

Shyamolina Ghosh, Lawrence W. Sheppard, Philip C. Reid, Daniel Reuman

Appendix

S1 The bound b **2**

List of Tables

S1 Notation used in the main text. 3

List of Figures

S1 Sampling sites on map for Plankton abundance data 4
S2 Position of 11 suction traps on map that sample flying aphids 5
S3 Planktons: C^n matrix plot for selected locations 6
S4 Aphids: C^n matrix plot for selected locations 7

S1 The bound b

In order to mitigate multiple-testing problems, we selected, prior to any analyses, one value of b for each dataset and used it. This is equivalent to selecting one statistic (the one which corresponds to the value if b we selected) for each dataset and using it. We do not present and did not compute results for other values of b, in order to reduce the chance that some values of b may produce "significant" results by chance alone. The values of b we selected for each dataset ($b = 1/2$ for the aphid data and $b = 1/3$ for the plankton data) were based on the lengths of the datasets, and past experience on the performance of the tail association statistics for datasets of a given length.

Table S1: Notation used in the main text.

Symbols	Definition
$s_i^n(t)$	Data (population or phenological) for species i in site n at time t.
cor_{l_b,u_b}	Partial Spearman correlation measuring association of two variables between the bounds l_b and u_b
C^n	Community tail association matrix for site n
D^n	Community-driver tail association matrix for site n
N_L^n	Number of species pairs at site n that showed stronger left- than right-tail associations. Equals the number of positive entries of C^n.
N_R^n	Number of species pairs at site n that showed stronger right- than left-tail associations. Equals the number of negative entries of C^n.
$A_{C,L}^n$	Sum of all positive entries of C^n
$A_{C,R}^n$	Sum of all negative entries of C^n
A_C^n	Total community tail association. Equals $A_{C,L}^n + A_{C,R}^n$, i.e., the sum of all non-NA entries of C^n.
$F_{C,L}^n$	A normalized analogue of $A_{C,L}^n$. Equal to $A_{C,L}^n/(A_{C,L}^n + \lvert A_{C,R}^n \rvert)$.
$F_{C,R}^n$	A normalized analogue of $A_{C,R}^n$. Equal to $A_{C,R}^n/(A_{C,L}^n + \lvert A_{C,R}^n \rvert)$.
A_D^n	Total community-driver tail association. Equals the sum of all non-NA entries of D^n.
$\alpha_C^n(i)$	Species-community tail association for species i. Equals the sum of non-NA entries of $C^n(i,j)$ over all j for which data were available.
$\alpha_D^n(i)$	Species-driver tail association. Equals the sum of non-NA entries of $D^n(i,k)$ over all k for which data were available.

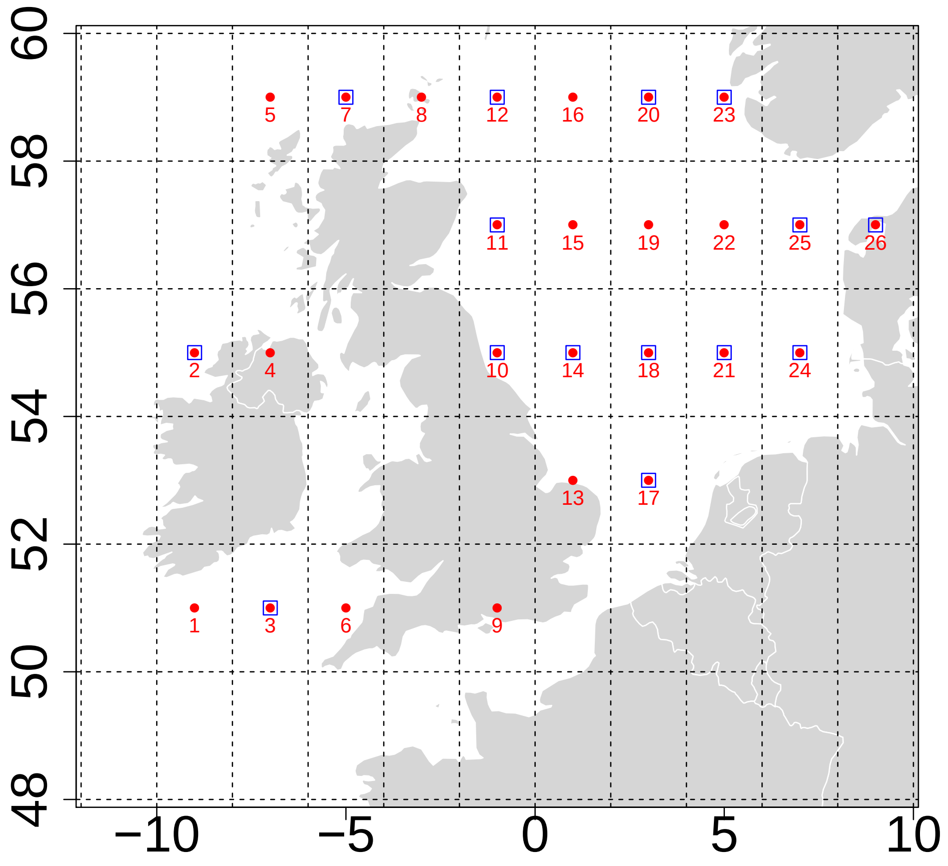

Figure S1: Plankton abundance data were obtained from 26 locations (2° × 2° grid cells) for the years 1958-2013. We analyzed data from 15 locations (blue boxes) based on criteria listed in the Data section of the main text.

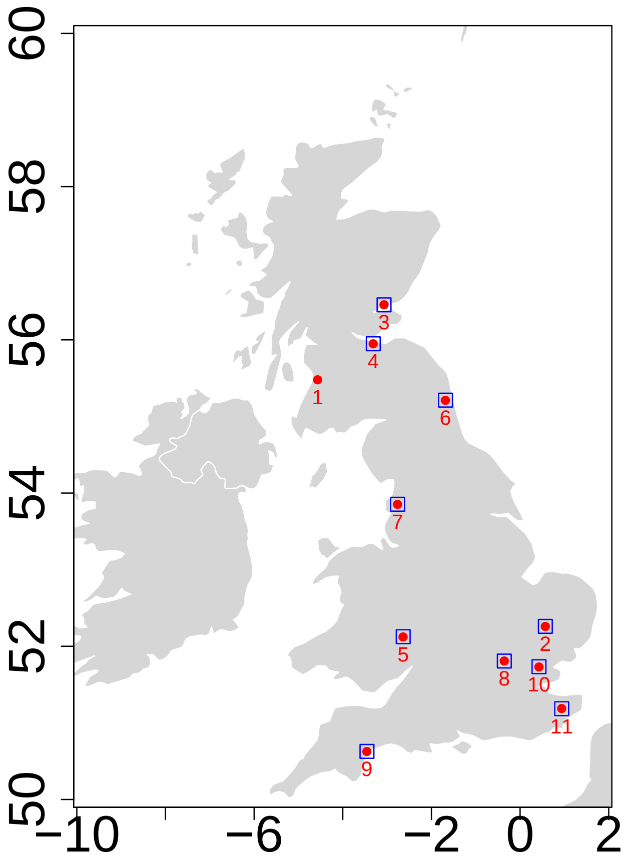

Figure S2: Aphid first flight data were obtained from 11 suction traps located across the UK for the years 1976-2010. We analyzed data from 10 of the traps (blue boxes) based on criteria listed in the Data section of the main text.

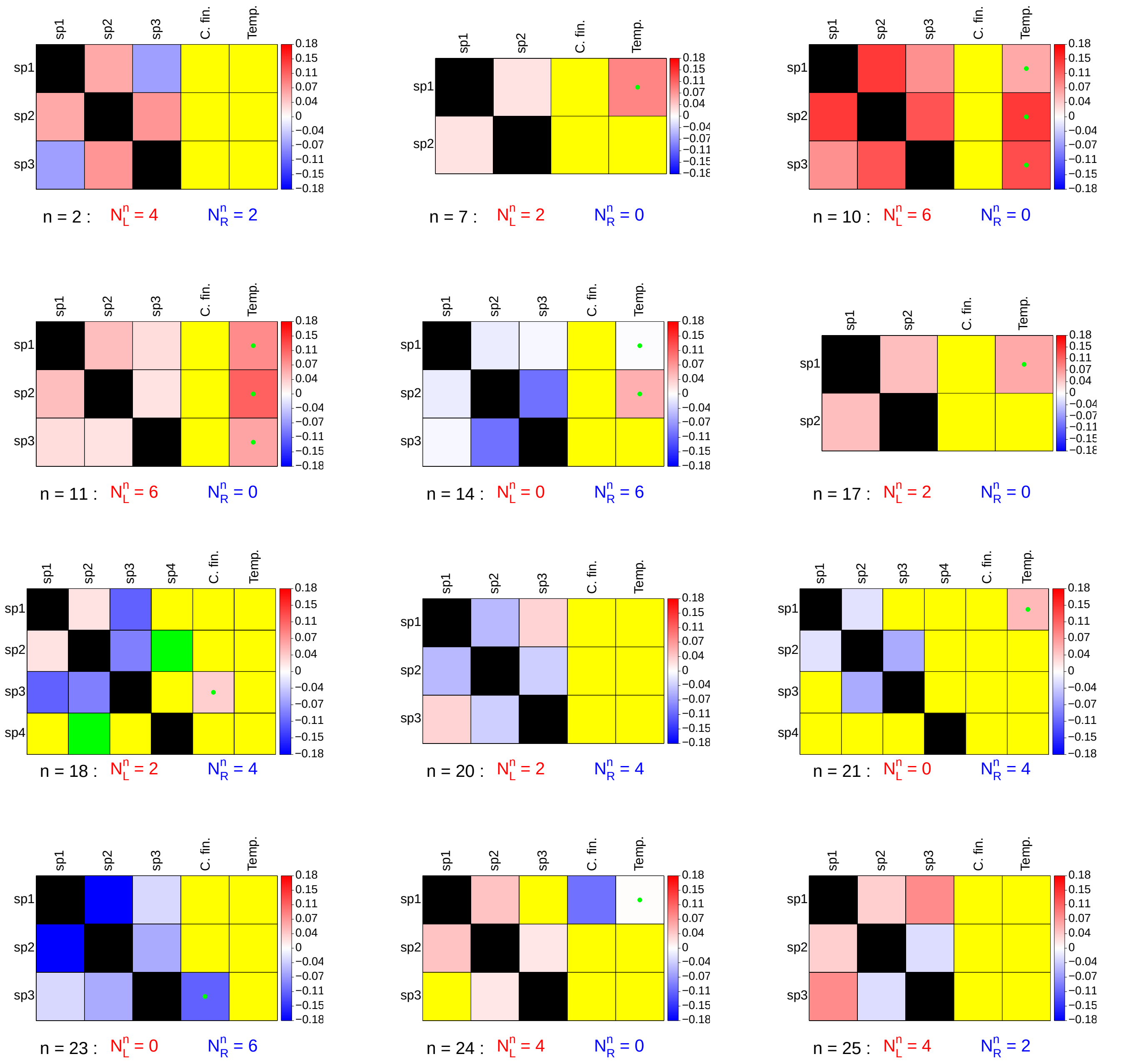

Figure S3: As Fig. 2A, B, but for other plankton sampling locations. Location 3 is not shown because the hypothesis that *Ceratium* times series were independent could not be rejected for that location (Methods). The two green squares for location 18 were the only two *Ceratium* time series for which the association was significantly negative. This association was not considered for our analyses, which focussed on synchronous dynamics (see Introduction).

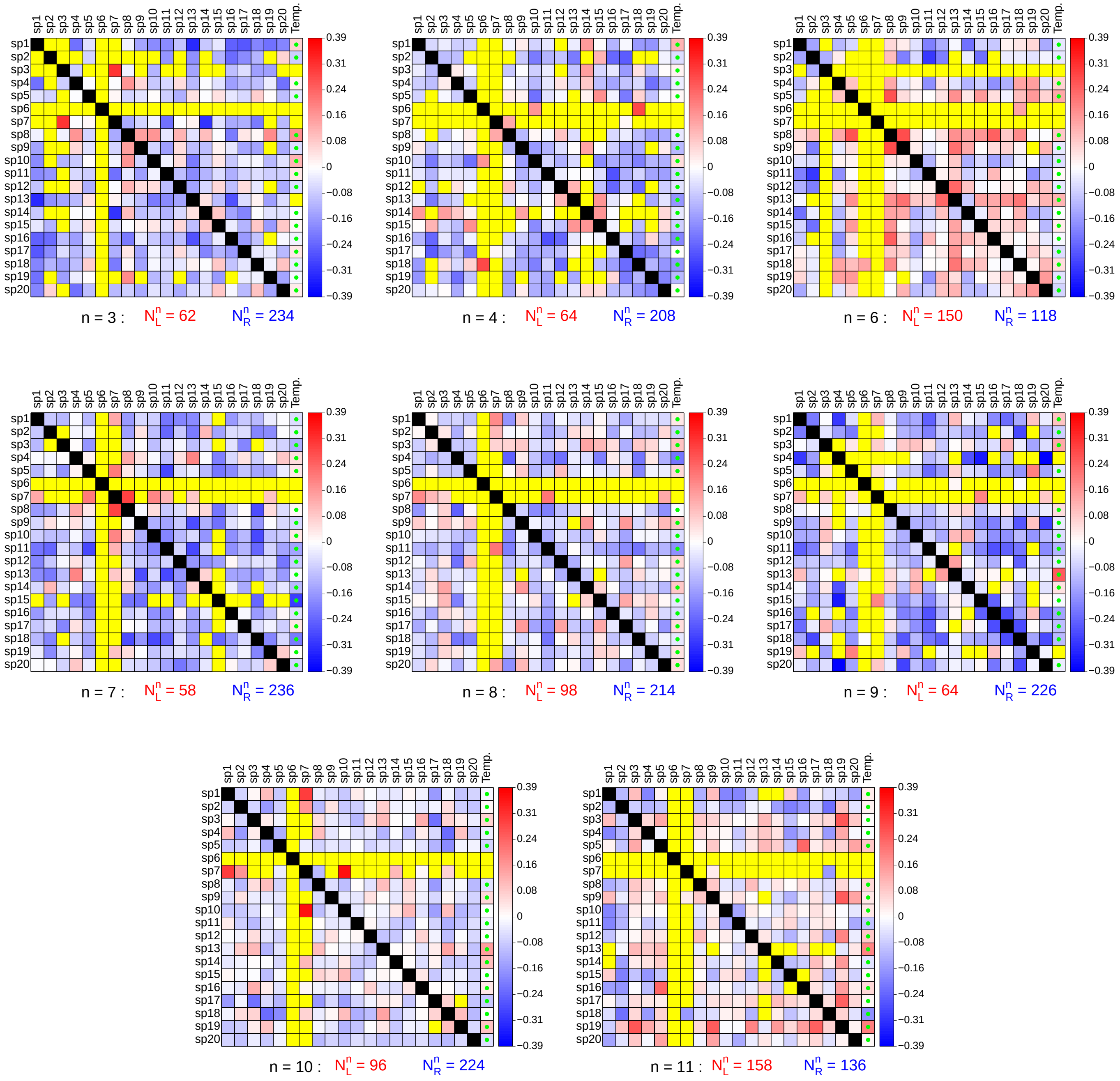

Figure S4: As Fig. 3A, B, but for other aphid sampling locations.
